# Septo-optic dysplasia caused by a novel *FLNA* splice site mutation: a case report

**DOI:** 10.1186/s12881-019-0844-5

**Published:** 2019-06-24

**Authors:** A. Fernández-Marmiesse, M. S. Pérez-Poyato, A. Fontalba, E. Marco de Lucas, M. T. Martínez, M. J. Cabero Pérez, M. L. Couce

**Affiliations:** 1 Unit for the Diagnosis and Treatment of Congenital Metabolic Diseases, Clinical University Hospital of Santiago de Compostela, Health Research Institute of Santiago de Compostela, Santiago de Compostela, Galicia Spain; 2 Pediatric Neurology Unit, Department of Pediatrics, Marqués de Valdecilla University Hospital, Santander, Cantabria Spain; 3 Department of Genetics, Marqués de Valdecilla University Hospital, Santander, Cantabria Spain; 4 Department of Radiology, Marqués de Valdecilla University Hospital, Santander, Cantabria Spain

**Keywords:** FLNA, Septo-optic dysplasia

## Abstract

**Background:**

Septo-optic dysplasia (SOD), also known as de-Morsier syndrome, is a rare disorder characterized by any combination of optic nerve hypoplasia, pituitary gland hypoplasia, and midline abnormalities of the brain including absence of the septum pellucidum and corpus callosum dysgenesis. The variable presentation of SOD includes visual, neurologic, and/or hypothalamic-pituitary endocrine defects. The unclear aetiology of a large proportion of SOD cases underscores the importance of identifying novel SOD-associated genes.

**Case presentation:**

To identify the disease-causing gene in a male infant with neonatal hypoglycaemia, dysmorphic features, and hypoplasia of the optic nerve and corpus callosum, we designed a targeted next-generation sequencing panel for brain morphogenesis defects. We identified a novel hemizygous deletion, c.6355 + 4_6355 + 5delAG, in intron 38 of the *FLNA* gene that the patient had inherited from his mother. cDNA studies showed that this variant results in the production of 3 aberrant *FLNA* transcripts, the most abundant of which results in retention of intron 38 of *FLNA*.

**Conclusions:**

We report for the first time a case of early-onset SOD associated with a mutation in the *FLNA gene*. This finding broadens the spectrum of genetic causes of this rare disorder and expands the phenotypic spectrum of the *FLNA* gene.

## Background

Septo-optic dysplasia (SOD), also known as de-Morsier syndrome (OMIM # 182230), is defined as any combination of the following: (1) optic nerve hypoplasia (ONH); (2) midline neuro-radiological abnormalities such as agenesis of the corpus callosum and absence of the septum pellucidum; and (3) pituitary hypoplasia with consequent panhypopituitarism [1]. Approximately 30% of all cases are characterized by the presence of all 3 alterations, while hypopituitarism and absent septum pellucidum are observed in 62 and 60% of cases, respectively [1]. SOD is a rare congenital anomaly with a reported incidence of 1/10,000 and is equally prevalent in males and females [1].

SOD represents a clinical spectrum rather than a specific entity [2]. Affected patients may present during infancy with systemic alterations including neonatal hypoglycaemia, jaundice, seizures, failure to thrive, developmental delay and microgenitalia. Several ophthalmic manifestations are also described, including visual impairment, nystagmus, strabismus, and, occasionally, refractive errors.

The majority of cases are sporadic. In rare cases a familial history can be identified. Studies of familial cases point to a mutation in a developmental gene (*HESX1, SOX2, SOX3*) [3, 4]. In this case report, we present the first evidence implicating a mutation in the *FLNA* gene in early-onset SOD.

## Case presentation

Our patient is a boy born in 2015, the second child of unrelated, healthy white parents. He has a healthy older sister who was born in 2011. He was born at 37^6/7^ weeks gestation and delivered by vaginal birth (weight, 2.4 kg; z-score, − 1,65; length, 45 cm; z- score, − 2.45; head circumference, 31 cm; z-score, − 1.84; Apgar scores, 8 at 1 min and 8 at 5 min). Five hours after birth he was admitted to the neonatal unit with hypoglycaemia (point-of-care, 29 mg/dl) and received intravenous glucose infusion for 72 h. The results of cerebral ultrasound and laboratory tests for congenital cytomegalovirus infection were normal.

An initial evaluation at 6 months of age revealed global developmental delay. The patient’s build, nourishment, and stature were normal for his age (z-score, + 0.64). Examination revealed microcephaly (z-score, − 1.56), dysmorphic features (thin upper lip, ogival palate, low hair implantation, long philtrum, extensive palpebral fissures), limb anomalies (implantation anomalies of the right toes and the plantar pads on both feet), microphallus, and undescended testes.

**Fig. 1:**
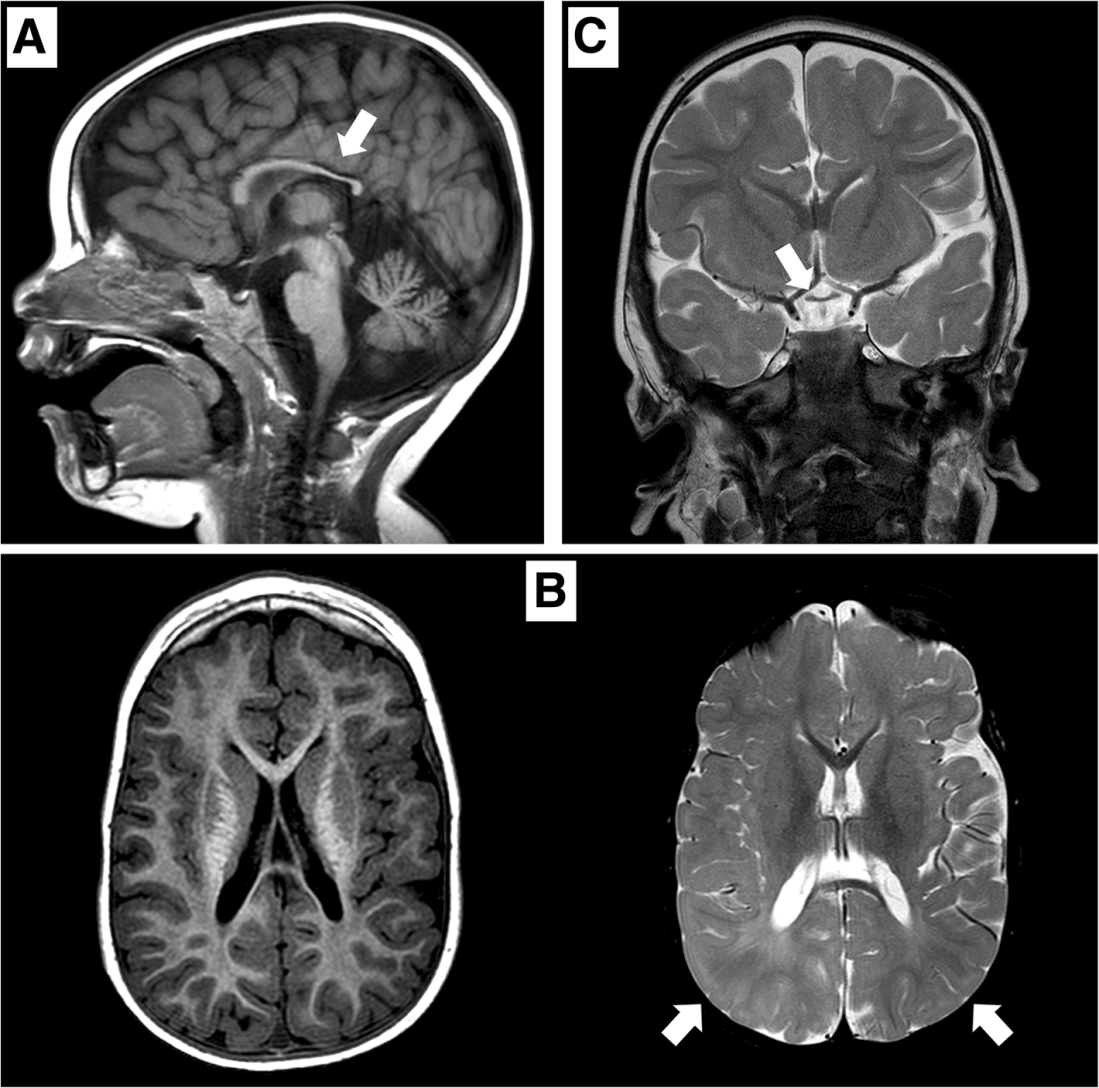
**a** Sagittal T1-weighted image showing hypoplasia of the corpus callosum (white arrow). **b** Axial T1-weighted (left) and axial T2-weighted (right) images showing hypoplasia of the corpus callosum and altered, poorly-defined signal in the bilateral subcortical parietal white matter (white arrows). **c** Coronal T2-weighted image showing hypoplasia of the optic chiasm (white arrow)

The patient presented generalized hypotonia; he was unable to achieve head control and showed horizontal jerk nystagmus, ocular motility disturbances, and visual impairment. There was no history of seizures. Cranial magnetic resonance imaging (MRI) showed delayed myelination, hypoplasia of the corpus callosum (Fig. 1), and pituitary hypoplasia. The flash visual-evoked potential response was absent.

Two months later, the patient achieved head control, but was unable to sit unaided. The results of metabolic tests (lactate, pyruvate, creatine kinase, amino acids, organic acids, thiamine, riboflavin, free fatty acids, LCFA, biotinidase, carnitine, glutaric acid, 4-OH butyric acid, purines, and pyrimidines) were normal.

During the first 2 years of life the patient’s growth rate was normal but decreased progressively, with z-scores of + 5.79 (first year) and + 0.85 (second year). After 2 years of age (when growth hormone [GH] begins to play a more important role) the patient’s growth rate dropped markedly between 24 and 30 months (z-score, − 2.1) and between 30 and 36 months (z-score, − 5.05).

Analysis of endocrine status revealed that at baseline (without stimulus) the patient’s hormone levels were within the normal range for the thyroid (TSH, T4), growth (IGF1, IGFBP3) and gonadal (FSH, LH) axes.

At 2 years of age the patient was unable to maintain eye contact and had not developed language skills. He was unable to sit unaided until the age of 3. At this point, brain MRI showed bilateral optic nerve hypoplasia and confirmed hypoplasia of the corpus callosum (Fig. 1). An echocardiogram performed to evaluate congenital heart defects revealed interventricular septum hypertrophy without obstruction.

## Methods

### Next-generation sequencing analysis

Targeted enrichment was performed using in-solution hybridization technology (Sure Select XT; Agilent Technologies; Santa Clara, California) and the MiSeq platform (Illumina; San Diego, California) was used for subsequent sequencing. A custom Sure Select probe library was designed to capture the exons and exon-intron boundaries of 166 genes (list available on request). This list included genes associated with all forms of brain morphogenesis defects identified in searches of the Online Mendelian Inheritance in Man (OMIM) database and recently published studies. To ensure optimal representation of all regions of interest, we designed different subgroups of probes according to guanine-cytosine content and location with respect to repetitive sequences [5]. Sequence capture, enrichment, and elution were performed according to the manufacturer’s instructions. Captured fragments were sequenced in pair-end 100-base mode using the MiSeq platform. Image analysis and processing of fluorescence intensity in sequences (“base calling”) was performed using Real Time Analysis (RTA) software v.1.8.70 (Illumina), and the FastQC v0.10.1 program [6] was used for data quality control. Reads were aligned to the reference genome GRCh37 with BWA v0.7.9a [7]. NGSrich v0.7.5 software [8] was used for quality control prior to variant detection, and BEDTools 2.17.0 [9] and Picard 1.114 [10] for the intermediate steps. VarScan v.2.3.6 [11] and SAMtools v0.1.19 [12] software were used for variant detection and SnpEff v4.3 for variant annotation [13]. Prioritization was based on the frequency of variants (MAF < 0.01) in 1000 genomes, dbSNP 137, Exome Aggregation Consortium ExAC, the exome variant server (EVS), the Genome Aggregation Database (gnomAD), and our in-house population database. Possible variant pathogenicity was assessed using CONDEL [14], GERP++ [15], and Human Splicing Finder v.HSF3.0 [16].

To rule out the presence of other possible variants that could potentially mimic the patient’s phenotype, we performed a wider scope panel (Neuroexome), which sequenced a total of 1860 genes (list available on request) implicated in rare metabolic and neurological diseases. We detected no variants that could be potentially associated with our patient’s phenotype.

### mRNA expression studies

Total RNA was extracted from T-cells and reverse-transcribed using M-MLV Reverse Transcriptase (Invitrogen, San Diego, CA, USA). The resulting cDNA was amplified by PCR using primer pairs (forward, 5′- CAAGGTGGACATCAACACAG-3′; reverse, 5′- GTCACCTGGGCTGTCATAT-3′) corresponding to sequences located in exons 38 and 40 of the *FLNA* gene. PCR fragments were separated by agarose gel electrophoresis and sequenced by Sanger sequencing.

## Results

**Fig. 2:**
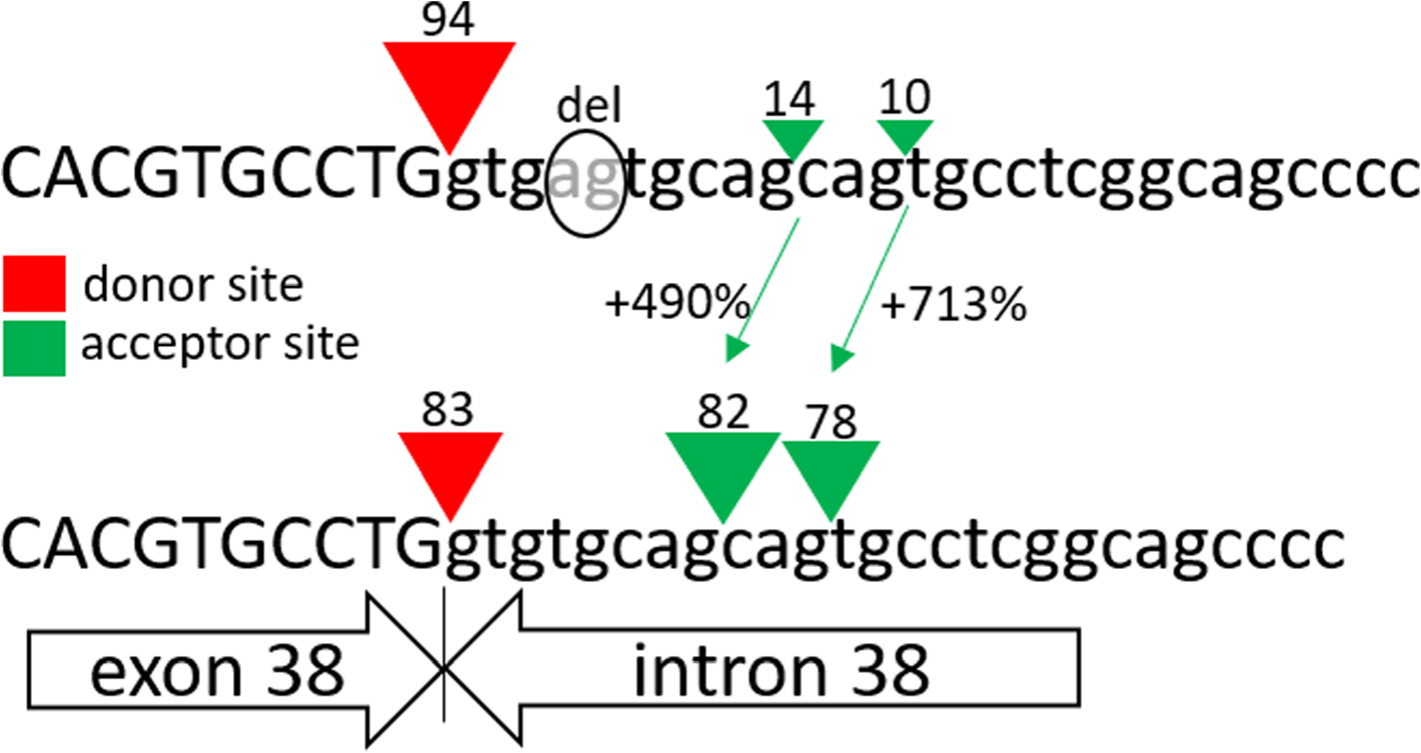
Results produced by the Human Splicing Finder prediction program (HSF 3.0) for the patient’s intronic variant. The 2-bp deletion is strongly predicted to give rise to 2 novel acceptor sites situated very close to the wild type donor site

Next-generation sequencing detected a rare intronic variant c.6355 + 4_6355 + 5delAG in hemizygous state in the *FLNA* gene (reference sequence NM_001456.3). This variant was not present in any public variant databases, nor in any of the 1084 chromosomes from our in-house database. The variant consists of the deletion of 2 nucleotides in positions + 4 and + 5 of intron 38. Given its location in a region that is potentially sensitive to splicing, in silico analysis was performed using Human Splicing Finder (HSF v3.0) software to evaluate the possible effect of the variant on *FLNA* mRNA splicing. The results of the in silico analysis indicate that 2 powerful exonic acceptor splice sites created at the beginning of intron 38 of *FLNA* likely hinder correct splicing (Fig. 2). This explains the retention of intron 38 (or part thereof) in the 3 mutant transcripts.

The patient’s variant was inherited, and it was identified as a de novo mutation in the patient’s mother. The patient’s sister is a heterozygous carrier of the variant.

**Fig. 3:**
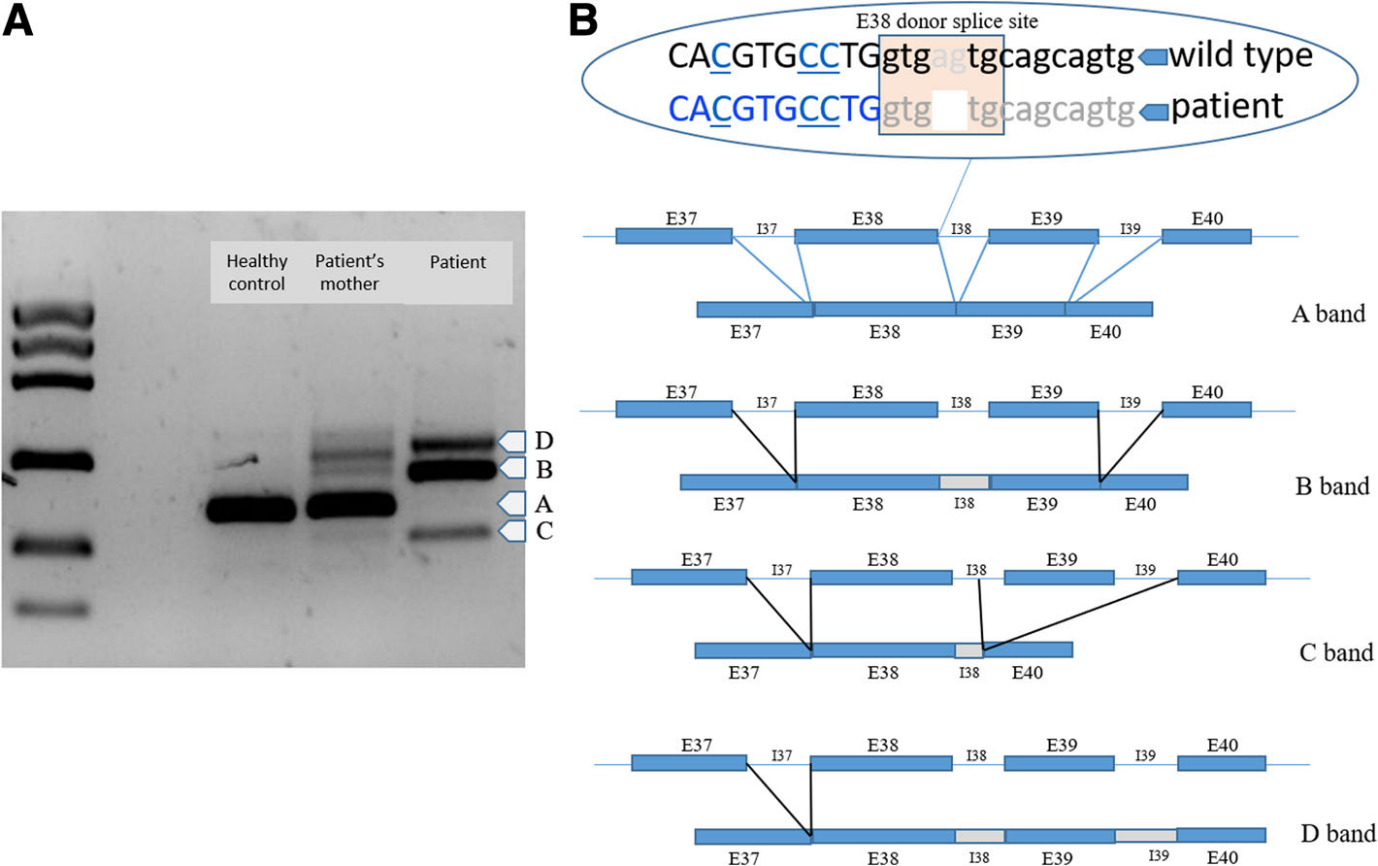
**a** Electrophoresis of cDNAs from the *FLNA* genes of a healthy control, the patient’s mother (heterozygous for the *FLNA* variant), and the patient (hemizygous). **b** Different FLNA splicing isoforms found in the patient: the A-band corresponds to wild type cDNA, the B-band includes intron 38; the C-band includes a portion of intron 38 but not exon 39; and the D-band includes introns 38 and 39

cDNA studies were conducted to determine whether the variant affected splicing. RT-PCR amplification revealed the total absence of wild type cDNA in the patient (A band) and 3 mutated cDNAs, all of which appear to give rise to a premature stop codon. In the most abundant mutant transcript (B band) intron 38 is retained while in the longest transcript (D band) introns 38 and 39 are retained (Fig. 3). In the smallest cDNA (C band) approximately half of intron 38 is present but exon 39 is absent. cDNA analysis of the patient’s mother revealed the presence of wild type mRNA, as well as the same 3 mutated transcripts found in the patient, albeit to a lesser extent. cDNA PCR was performed under different conditions to determine whether there was any variation in the PCR products obtained. In all cases similar results were obtained.

## Discussion & Conclusions

SOD-associated mutations have been identified in genes that encode transcription factors essential for normal forebrain/pituitary development, such as *HESX1*, *SOX2*, *SOX3*, *OTX2*, *TCF7L1* or *TAX1BP3* implicated in regulation of the Wnt/β-catenin signaling pathway [4, 17–23]. Mutations in genes implicated in Kallmann syndrome have also been recently described in SOD patients. These include fibroblast growth factor receptor 1 (*FGFR1*), the ligand *FGF8*, and *PROKR2* [24–26], all of which are involved in the maturation of gonadotrophin-releasing hormone (GnRH) neurons and/or their migration from the olfactory placode to the ventral forebrain [27, 28]. Nonetheless, the aetiology of SOD remains unclear in the majority of patients.

The X-linked *FLNA* gene encodes filamin A, a non-muscle actin-binding protein that serves as a molecular scaffold to transduce various receptor and intracellular signals throughout the actin cytoskeleton. It is required for locomotion of many cell types. It is expressed at high levels in the developing cortex, where it contributes to neuronal migration to the cortex, and is also essential for embryogenesis [29–37]. As described for many other X-linked genes, there are significant phenotypic differences between males and females with *FLNA* mutations. Heterozygous truncating mutations in *FLNA* result in periventricular nodular heterotopia (PVNH) as the principal presenting feature, a disorder in which the migration of neurons to the cerebral cortex is disrupted and neurons persist as nodules lining the ventricular surface. The majority of patients with *FLNA*-associated PVNH are female, since truncating variants are usually lethal in males [38]. Surviving males with truncating *FLNA* variants usually have distal or mosaic mutations, whereby some residual protein function is retained [39–44]. Females with *FLNA-*associated PVNH present with seizures and, occasionally, subtle learning disabilities [39], as well as other clinical signs including vascular and cardiac defects and congenital intestinal pseudo-obstruction (CIPO), which is characterized by anomalous intestinal smooth muscle layering. Some *FLNA-*associated PVNH patients show variable soft connective tissue involvement as well as X-linked cardiac valvular dystrophy (XCVD), giving rise to a phenotype known as Ehlers-Danlos syndrome (EDS) with periventricular heterotopia [45–50]. In a subset of male patients, the clinical presentation is characterized by PVNH accompanied by cardiovascular malformations and/or CIPO [51, 52]. The mechanisms underlying this phenotypic variability remain unclear.

Localized gain-of-function variants in *FLNA* lead to otopalatodigital (OPD) spectrum disorders, characterized primarily by skeletal dysplasia including otopalatodigital syndrome types I (OPD1) and II (OPD2), Melnick-Needles syndrome (MNS), frontometaphyseal dysplasia (FMD1) and terminal osseous dysplasia (TODPD), affecting both males and females (except for TODPD which has only been described in females) [53–57]. Several of these mutations are recurrent, and all are clustered in 4 regions of the gene: the actin-binding domain and rod domain repeats 3, 10, and 14/15. The patterns of mutation, X-chromosome inactivation, and phenotypic manifestations in this class of mutations indicate gain-of-function effects, implicating filamin A in signalling pathways that mediate organogenesis in multiple systems during embryonic development.

Our patient was diagnosed with SOD based on the presence of 2 out of 3 of the classic triad features (ONH, pituitary hormone abnormalities, and midline brain defects). He exhibits bilateral optic nerve hypoplasia, ocular motility disturbances, and midline brain defects with neonatal hypoglycaemia, but no clear endocrinological defects to date.

The patient carries a hemizygous splicing mutation inherited from his mother, in whom this variant arose as a consequence of a de novo event. This explains the lack of other cases in the patient’s family as well as the deleterious nature of the mutation. The results of our in-silico variant evaluation and mRNA studies indicate that the mutation affects *FLNA* splicing. PCR amplification revealed three mutant FLNA transcripts and the total absence of wild type cDNA. In the most abundant mutant transcript (B band) intron 38 is retained and a premature stop codon emerges in exon 39. In the longest transcript (D band), in which introns 38 and 39 are retained, a stop codon emerges in intron 38. In the smallest cDNA (C band), in which approximately half of intron 38 is present but exon 39 is skipped, a stop codon appears in exon 40. cDNA analysis of the patient’s mother revealed the presence of wild type mRNA, as well as the 3 same mutated transcripts found in the patient, albeit to a lesser extent, which probably explains the absence of disease in both the mother and sister. The survival of the patient suggests that the novel transcripts generated by this variant retain some residual protein function. Our patient also presents interventricular septum hypertrophy and limb anomalies, also characteristic of patients with *FLNA* mutations. Taken together, these findings strongly suggest that our patient’s variant is implicated in the clinical picture. Nonetheless, further functional studies will need to be performed in order to better elucidate the effect of this mutation.

In summary, we describe for the first time a case of SOD caused by an underlying *FLNA* mutation. This finding broadens the spectrum of genetic causes of this complex pathology and expands the phenotypic spectrum of *FLNA* gene mutations. Early diagnosis will be useful to provide prospective parents with genetic counselling regarding future pregnancies.

## Data Availability

The principal data generated and/or analyzed in this study are included in the published article. The corresponding datasets are available from the corresponding author on request.

## Abbreviations

ACTH: Adrenocorticotropic hormone
cDNA: Complementary DNA
CIPO: Congenital intestinal pseudo-obstruction
EDS: Ehlers-Danlos syndrome
LCFA: Long chain fatty acid
MRI: Magnetic resonance imaging
ONH: Optic nerve hypoplasia
OPD: Otopalatodigital
PCR: Polymerase chain reaction
PVNH: Periventricular nodular heterotopia
RT-PCR: Reverse transcription polymerase chain reaction
SD: Standard deviation
SOD: Septo-optic dysplasia
TODPD: Terminal osseous dysplasia with pigmentary defects
XCVD: X-linked cardiac valvular dystrophy
